# Successful Use of Ectopic Pelvic Kidney for Living Related Donation Technical Aspects and Literature Review

**DOI:** 10.1155/2017/8286257

**Published:** 2017-06-04

**Authors:** R. C. Minnee, H. J. A. N. Kimenai, J. van de Wetering, J. N. M. Ijzermans

**Affiliations:** ^1^Department of Surgery, Division of HPB & Transplant Surgery, Erasmus University Medical Center (Erasmus MC), Rotterdam, Netherlands; ^2^Department of Nephrology, Erasmus University Medical Center (Erasmus MC), Rotterdam, Netherlands

## Abstract

Ectopic pelvic kidneys can provide an additional source of organs for transplantation. They are often excluded from donation in living donation programs mainly due to aberrant vascular and urinary anatomies. We present a donor with an ectopic left kidney, who successfully donated his kidney. The use of ectopic pelvic kidney for living kidney transplantation is a highly demanding surgical procedure but after extensive preoperative investigation in high volume centers with surgical expertise in vascular reconstruction and access surgery, ectopic pelvic kidneys should not be a contraindication for donation and should be considered as a viable option.

## 1. Introduction

Live donor kidney transplantation is the optimal method of renal replacement therapy for most patients with end stage renal disease in terms of short- and long-term results [1]. Shortage of available donor kidneys compels us to expand the supply of living donor kidneys. Essentially, the donor kidney must be anatomically and functionally suitable for transplantation, and the donor must be in good medical and psychological health and left with a good renal function from the remaining kidney.

The use of ectopic pelvic kidneys can provide an additional source of organs for transplantation.

Ectopic pelvic kidney is a relatively rare congenital anomaly in which the kidney fails to ascend to its lumbar position during embryonic development. The incidence of pelvic kidneys is reported as 1 in 1000 autopsies; only routine ultrasound screening in children revealed a lower incidence of 1 in 5000 [2, 3]. The location of an ectopic kidney can be pelvic, lumbar, abdominal, and thoracic or crossed fused. Ectopic pelvic kidney slightly occurs more on the left side with predominance in male subjects. Due to malrotation, the ectopic pelvic kidney might have a flattened, discoid shape. This incomplete rotation leads to an undeveloped renal pelvis with a variety of congenital anatomies such as a shorter ureter, defective ureteral drainage, and multiple renal arteries and renal veins. Ectopic pelvic kidneys are often excluded from donation in living donation programs mainly due to these aberrant vascular and urinary anatomies resulting in only a few successful case reports [4–15]. We advocate the use of these kidneys for transplantation in high volume transplant centers and present a successful living-unrelated kidney transplantation of an ectopic pelvic kidney; in addition, we present a review of the literature to focus on the technical aspects.

## 2. Case Report

**Figure 1:**
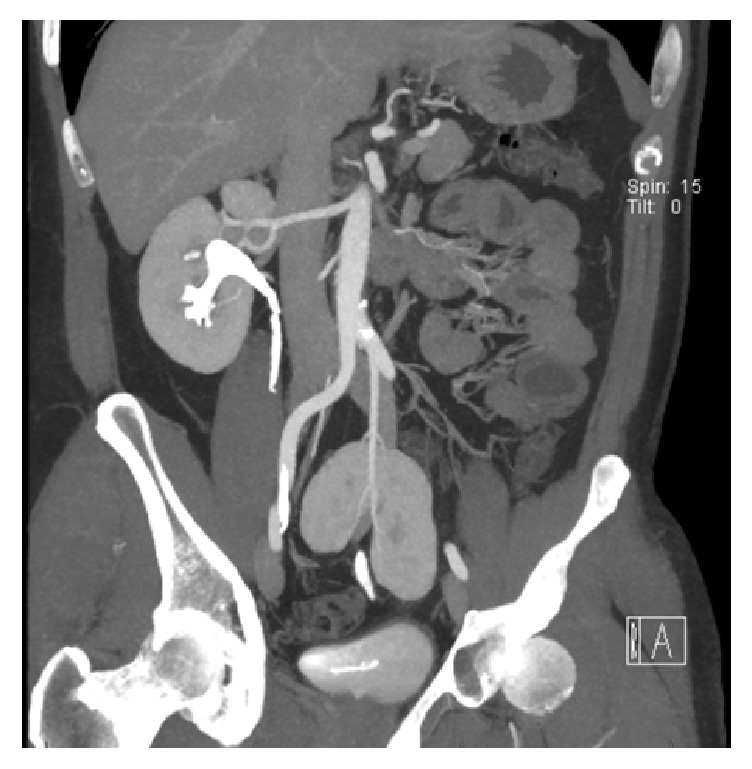
CT scan with coronal image of ectopic left pelvic kidney showing the middle polar artery.

**Figure 2:**
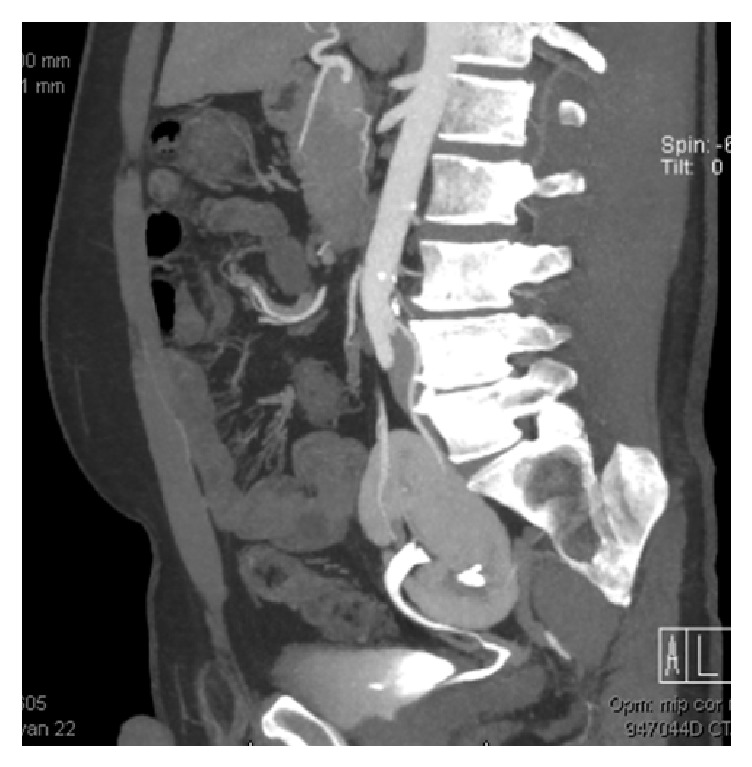
CT scan with sagittal image of ectopic left pelvic kidney showing the upper and middle polar artery.

The donor was a 43-year-old man donating a kidney to his wife. In the past, he underwent arthroscopic meniscus repair on his left knee and micro discectomy of a herniated nucleus pulposus. He did not use any medication and reported no allergies. During workup, an ectopic left kidney and a normally placed right kidney were found. Physical examination was normal with a BMI of 25.7. Blood and urine examination revealed no abnormalities. His measured creatinine clearance was 123 mL/min. The computed tomography (CT) angiogram performed showed 1 artery, 1 vein, and 1 ureter of the left kidney (Figures 1 and 2). The ectopic left kidney had a relative function of 40% in conventional technetium-99m (Tc-99m) mercaptoacetyltriglycine (MAG3) scan. During the renal transplant multidisciplinary meeting, it was recommended to select the left kidney for donation.

**Figure 3:**
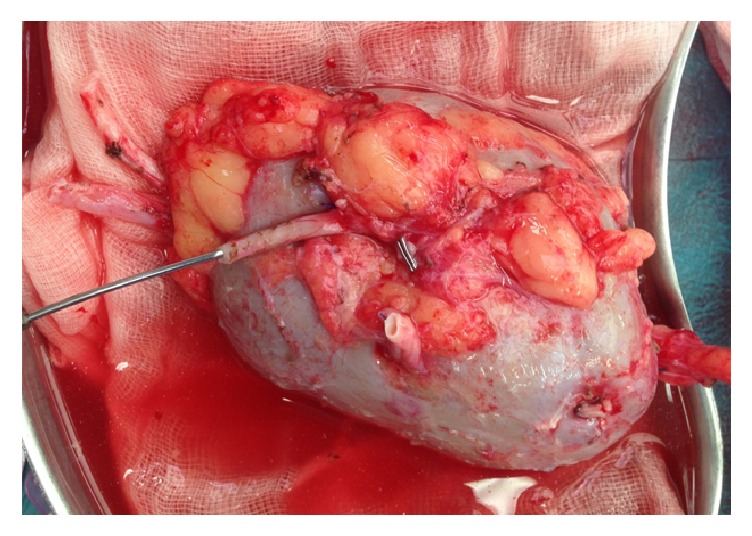
Left pelvic kidney with additional upper polar artery and an additional middle polar artery before reconstruction.

**Figure 4:**
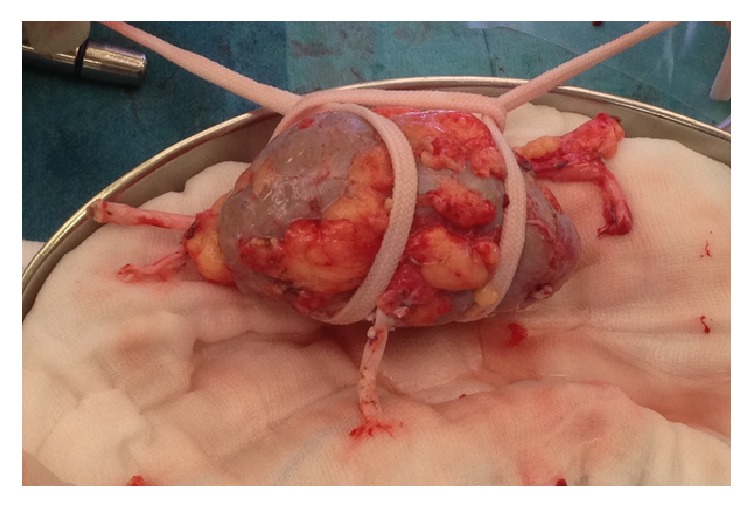
After end-to-side reconstruction of the middle polar artery and main renal artery.

A left open donor nephrectomy was performed through a Gibson incision. The donor kidney had additional blood vessels undetected on CT scan. There were an additional upper polar artery and an additional middle polar artery (Figure 3). On the bench, an end-to-side anastomosis was made between the middle polar artery and the main renal artery with Prolene 7.0 (Figure 4).

The recipient was the donor's 43-year-old wife with end stage renal failure secondary to tubulointerstitial nephritis. The donor kidney was placed in the right iliac fossa. The renal vein was anastomosed to the common iliac vein, whereas the reconstructed renal artery was anastomosed end to side to the external iliac artery and the upper polar artery to the common iliac artery. The anastomosis time for all anastomoses was 32 minutes. The method used to establish urinary continuity was extravesical ureteroneocystostomy and closure of the abdominal wall in layers. The ureteroneocystostomy was stented with an externally draining 8-French catheter for 9 days. According to local protocol, a standard dose of 12.000 U heparin daily was given during the first 5 days due to the arterial reconstruction. The postoperative course of the donor and the patient was uneventful.

## 3. Discussion

The use of an ectopic kidney for transplantation in a living transplant program has been only reported in thirteen patients (Table 1) [4–15]. In all cases, extensive investigation of live donors was carried out. The majority of ectopic kidneys had multiple renal vessels especially renal arteries (77%). However, despite comprehensive investigation in 4 cases (29%) (including our case), discrepancy was found between the preoperative imaging and operative findings [4, 11, 14]. In all 4 cases, additional renal veins and/or arteries were visualized. So, even after extensive preoperative imaging, a cautious approach should be undertaken during the donor nephrectomy for the existence of additional vascular structures.

Several studies reported clinicoradiological discrepancies between preoperative CT renal angiography and operative findings ranging from 5% to 15% [16–18]. Retrospectively, Johnson et al. could not identify 3 of the 12 discrepancies between CT angiographic and operative findings on image review [16]. This reveals an effect of experience on reporting accuracy and greater awareness of potential reporting pitfalls, which was the case in our report.

All kidney grafts showed immediate graft function except in one case requiring two hemodialysis sessions (Table 2). All arterial and venous reconstructions were flawless, showing that reconstruction of multiple renal vessels in ectopic pelvic kidneys is feasible for transplantation [4, 11, 13–15].

Ureteral duplication occurs in approximately 0.8% of the general population and the incidence of duplicated ureters in ectopic pelvic kidney is unknown [19]. Approximately 50% of ectopic kidneys have a hydronephrosis. Half of these cases are due to obstruction of the ureteropelvic or the ureterovesical junction (70% and 30%, resp.), 25% from reflux grade III or greater, and 25% from the malrotation alone [20]. Despite all aberrant urinary anatomies described in the literature, all transplanted ectopic pelvic kidneys reported in the literature had a normal single ureter and urinary collecting system. When the ureter is very short, ureteroureterostomy or pyelovesicostomy is an alternative option.

Laparoscopic donor nephrectomy has been performed in 2 cases [8, 12]. Key points for laparoscopic donor nephrectomy are careful interpretation of the preoperative imaging and use of a Endo Retract™ articulating fan retractor to increase the working space [12].

Hence, these donors should have extensive investigations of the renal vasculature and anatomy variation using computed tomography angiography or magnetic resonance angiography. Intravenous pyelography can also be performed to detect urinary tract abnormality and, additionally, nuclear renography is necessary to assess the renal function of each kidney.

The main limitation in all the case reports is the presence of publication bias. Only successful transplantations are most likely reported and published.

The use of ectopic pelvic kidney for living kidney transplantation is a highly demanding surgical procedure but nonetheless a feasible one. After extensive preoperative investigation in high volume centers with surgical expertise in vascular reconstruction and access surgery, ectopic pelvic kidneys should not be a contraindication for donation and should be considered as a viable option. Since the presence of an ectopic pelvic kidney is a relatively common condition, it must be noted that the use of these kidneys may expand the donor pool in experienced hands.

## Figures and Tables

**Table 1:** 

Author	Number of patients	Donor-recipient relationship	Kidney	Number of arteries^*∗*^	Number of veins^*∗*^	Number of ureters^*∗*^	Number of arteries^#^	Number of veins^#^	Number of ureters^#^	Discrepancy imaging versus surgery
Kim 1973	1	Sister to brother	Left	1	1	1	1	1	1	N
Spanos 1976	2	Mother to son	Right	3	1	1	3	1	1	N
		Brother to brother	Right	2	1	1	2	1	1	N
Bacharach 1984	1	Brother to brother	Right	2	1	1	3	1	1	Y
Luke 2003	1	Uncle to niece	Left	2	2	1	2	2	1	N
Grogan 2004	1	Son to mother	Right	1	1	1	1	1	1	N
Boughey 2004	1	Father to son	Right	3	1	1	3	2	1	Y
Li 2006	1	Unrelated	Right	3	3	1	3	3	1	N
Papanikolaou 2007	1	Mother to son	Left	1	1	1	1	1	1	N
Goldsmith 2009	1	Father to son	Left	2	1	1	3	2	1	Y
He 2012	1	Wife to husband	Left	2	2	1	2	2	1	N
Yaich 2014	1	Son to father	Left	3	1	1	3	1	1	N
Siemens 2015	1	Father to son	Left	2	1	1	2	1	1	N

^*∗*^Number of arteries/veins and ureters on preoperative imaging; ^#^number of arteries/veins and ureters during explantation.

**Table 2:** 

Author	Number of patients	AT	Vascular reconstruction	Vascular reconstruction	Postoperative course	Graft function
Kim 1973	1	N/A	N	—	Uneventful	Immediate
Spanos 1976	2	N/A	N	2 arteries ETS, smallest on internal iliac artery	Uneventful	Immediate
		N/A	N	No reconstruction, smallest artery ligated	Uneventful	Immediate
Bacharach 1984	1	N/A	Y	2 arteries STS, smallest artery ligated	Uneventful	Immediate
Luke 2003	1	32 min	Y	2 arteries STS, 2 renal veins ETS	Uneventful	Immediate
Grogan 2004	1	N/A	N	—	Uneventful	Immediate
Boughey 2004	1	N/A	Y	2 arteries STS, 2 renal veins ETS	Uneventful	Immediate
Li 2006	1	N/A	Y	2 arteries STS, smallest on internal iliac artery, 3 renal veins reconstructed with vein interponate	Uneventful	Immediate
Papanikolaou 2007	1	N/A	N	—	Uneventful	Immediate
Goldsmith 2009	1	50 min	Y	2 arteries ETS, 1 artery ETE on epigastric artery with vein interponate, 2 renal veins ETS	Uneventful	Immediate
He 2012	1	45 min	N	1 artery ETS, 1 artery ETE on internal iliac artery, 2 renal veins ETS	Uneventful, 6 weeks later lymphocele followed by surgery	Immediate
Yaich 2014	1	N/A	N	1 artery ETS, 2 small arteries ligated	Uneventful	Immediate
Siemens 2015	1	N/A	N	2 arteries ETS	Acute vascular rejection	2 HD sessions

N/A: not applicable; ETS: end-to-site anastomosis; STS: side-to-side anastomosis; ETE: end-to-end anastomosis; AT: anastomosis time.
